# What Is Homœopathy?

**Published:** 1883-05

**Authors:** 


### WHAT IS HOMŒOPATHY?

This question can be fairly and briefly answered from its famous founder's own stand-point.

The system of Hahneman as he left it, may be comprehended in three fundamental maxims: First. Disease is not perverted vital action, or simply perverted physilogical processes of mind or body, but is entirely foreign to both—a possession from without, like the demoniac invasions of men's bodies and minds in the days of Christ.

Second. No disease, whatever its nature many be, can be diminished or cured, except by setting up another in its place.

Third. Dissimilar diseases do not cure or dominate each other.

Fourth. Similar diseases, alone, cure each other. "*Similia similibus curantur.*"

Hence the name Homœopathy, given by the glad father to his strange progeny—omoios, like and pathos, affection.

The infinitely absured doctrine, that by infinite dilution or subdivision, the potency of drugs is infinitely multiplied, seems to have been an after-thought with Hahneman. It has certainly no expression in the name, and is now disclaimed by that part of the school in whom common sense dominate narrowness, bigotry and ignorance.

